# Anthocyanin Content of Crackers and Bread Made with Purple and Blue Wheat Varieties

**DOI:** 10.3390/molecules27217180

**Published:** 2022-10-24

**Authors:** Alyssa Francavilla, Iris J. Joye

**Affiliations:** Department of Food Science, University of Guelph, Guelph, ON N1G 2W1, Canada

**Keywords:** anthocyanins, wheat, cracker processing, bread processing

## Abstract

Purple and blue wheats contain anthocyanins in the outer layers of the wheat kernel, and therefore purple and blue wholemeals can be a source of anthocyanins when developing processed cereal products. However, cereal processing is anticipated to cause significant anthocyanin losses. In this study, the anthocyanin content of crackers and bread made from one purple and three blue wholemeals was measured during processing and after baking. LC-MS/MS was used to confirm the presence of anthocyanins, and to tentatively identify them. Mixing and baking steps significantly decreased the anthocyanin content, whereas resting and fermentation steps did not. Purple and blue wholemeal samples reacted differently, indicating that the starting anthocyanin content, localization and composition may have some impact on anthocyanin retention. Additionally, dough systems with decreased pH were more protective of anthocyanins during intermediate processing steps, as were high-temperature, short-time baking procedures. This research provides insights into the processing steps that cause significant anthocyanin losses, and proposes some modifications to formulation and processing conditions which can further reduce losses.

## 1. Introduction

Wheat (*Triticum aestivum*) is consumed in large quantities globally, second only to rice as the most consumed cereal in the human diet [1,2,3]. Although refined white flours are often preferred over whole-grain ingredients in cereal products, the consumption of wheat (typically red and white varieties), and specifically whole grains, may play a role in reducing the risk of chronic disease [3,4,5]. Whole-grain wheat constituents such as fibre, and secondary metabolites (e.g., phenolic compounds, and carotenoids), are thought to be responsible for the health promoting effects associated with whole-grain wheat consumption. Coloured (blue, purple, and black) wheat grains additionally contain anthocyanins in the outer layers of the wheat kernel, and may confer further health benefits to consumers due to these compounds. Anthocyanin pigments are a large group of water-soluble polyphenols, and are responsible for the red, purple, and blue colours of many fruits, vegetables, and grains [6,7,8,9,10]. Anthocyanins have demonstrated in vitro antioxidant potential, and consumption of foods high in anthocyanins has been linked to a lower risk of developing chronic diseases [6,11]. 

Since cereal foods are staple foods globally, coloured cereal grains rich in anthocyanins should be explored as a source of antioxidants in the human diet. However, it has been well established that anthocyanins are sensitive to environmental stresses including exposure to heat, light, pH changes, and high oxygen levels [9]. Therefore, the processing of coloured cereal-based bakery products could result in drastically reduced anthocyanin contents. Common wheat processing operations include baking (high temperature treatments), exposure to prolonged shear (mixing and kneading), and exposure to pH changes (chemical and biological fermentation). 

The impact of cereal processing on wheat endogenous anthocyanins has been studied in some bread [12,13,14,15,16], muffin [17], cookie [18], and pasta [19] applications. In general, research has shown that thermal processing of coloured cereal grains has a negative impact on the anthocyanin content of the resulting product. However, these studies largely report the impact of overall processing on the resultant anthocyanin content, rather than the impact of intermediate steps. However, the intermediate processing steps, and characteristics of processed cereal products are diverse and may have varying effects on the final anthocyanin content. Therefore, the influence of specific processing steps should also be investigated to gain insight into the fate of anthocyanins during cereal processing and to design strategies to retain higher anthocyanin levels in the final product. 

We thus monitored the anthocyanin content of one purple and three blue wheat varieties during cracker and bread processing. The chosen products encompass a range of processing conditions, including short/long resting times, high/low dough moisture contents, and high/low baking temperatures. The aim was therefore to investigate (1) the effect of each processing step on the anthocyanin content of the product, (2) the role of wheat variety/colour on the anthocyanin content of intermediate and final products, and (3) the different effect of cracker and bread processing on anthocyanin content. 

## 2. Results and Discussion 

### 2.1. Stability of Endogenous Wheat Anthocyanins during Cracker Processing 

**Figure 1:**
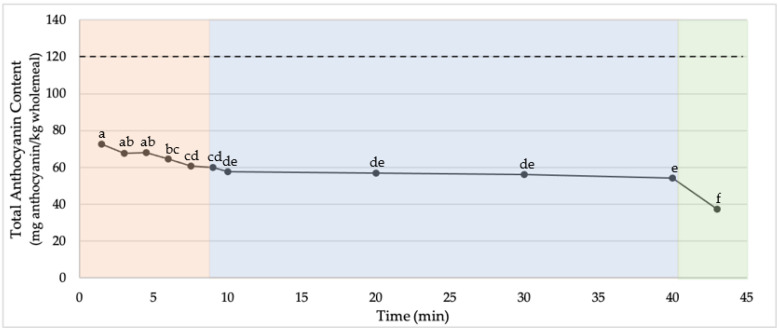
Total anthocyanin content (in mg anthocyanin/kg wholemeal) of AnthoGrain™ cracker dough, and crackers throughout the cracker production process (mixing = red, resting/shaping = blue, and baking = green). The dashed line indicates the starting anthocyanin content of the wholemeal used (samples with different letters are significantly different at α = 0.05).

Crackers were produced using wholemeal from one purple and three blue wheat varieties. The average total anthocyanin content (TAC) of crackers produced from blue wheat was higher than the TAC in crackers made with purple wheats (Table 1). The TAC in the baked cracker of Blue 1 was significantly higher than the values found for the other tested varieties. All of the cracker samples demonstrated a significant loss of anthocyanins over the cracker production process. The total processing time for the crackers produced using this method was approximately 45 min from the initiation of mixing, to a fully baked cracker, and the decrease in anthocyanin content is visualized throughout the entire process in Figure 1. It can be observed that a substantial decrease in TAC occurs in the first 1.5 min of mixing, and throughout the mixing and baking steps. In contrast the TAC is well maintained during the resting and shaping steps. 

The loss of anthocyanin content (%) from each cracker processing step is presented in Table 2. In general, mixing and baking caused the most substantial losses in anthocyanin content. The impact of these steps differed depending on the wheat colour. Several processes occur during the dough mixing stage, including dispersion of ingredients, hydration of the wholemeal, emulsification of fats and water, gluten development, air incorporation, and activation of leavening ingredients [20]. These processes are necessary to produce a workable cracker dough, but have a significant impact on the anthocyanins present in the wheat bran. The hydration of the wholemeal, and therefore the bran, could allow some of the water-soluble anthocyanins to be extracted from the bran matrix into the dough liquor phase, where they would be more vulnerable to stresses such as changes in pH (from the activation of the leavening agents), and exposure to oxygen (from the air incorporation) and other ingredients [20]. The continuous inclusion of air into the cracker dough during mixing is likely a key factor in the high anthocyanin losses observed during mixing. Oxygen plays a significant role in anthocyanin degradation processes, and can accelerate these processes through direct oxidative mechanisms, and by facilitating reactions catalyzed by oxidizing enzymes [21]. The resting and shaping processes did not have a significant impact on TAC. While these involve prolonged exposure to room temperature, and extensive handling, there is little change in the external conditions of the dough which may mediate any possible anthocyanin degradation. Baking, conversely, resulted in a significant decrease in TAC. Anthocyanins are very susceptible to thermal stress, and rapidly degrade when exposed to high temperatures [14,22].

There were significant differences in the TAC losses between crackers made with purple and blue wheats. In blue wheat, baking caused more anthocyanin loss than mixing/resting did, while in purple wheat the opposite was observed. The localization of anthocyanins within the wheat kernel (i.e., pericarp vs. aleurone) may have contributed to this result. Anthocyanins within the (more damaged) pericarp may be less protected from oxidative stress caused by the mixing process, whereas (the higher remaining level of) anthocyanins in the aleurone layer may be more susceptible to thermal stress. Despite the intermediate differences in anthocyanin losses, there was no significant difference in the overall loss between the purple wheat, and blue wheats 1 & 3, over the entire production process.

Discrepancies between the anthocyanin content and composition of the starting material, may also contribute to the observed differences in anthocyanin loss between the wheat varieties (e.g., the presence of anthocyanins with more methylation, and therefore greater stability). In isolated model systems, glycosylated cyanidin derivatives were shown to be more thermally stable than glycosylated delphinidin derivatives [22]. Similar observations were made in a cereal product. Blue wheat varieties have been shown to contain predominantly delphinidin-based anthocyanins, in comparison to purple wheat where cyanidin derivatives are more common [23,24,25,26,27]. In these wheat varieties, peonidin was another predominant aglycone found in both the blue and purple wheats. As expected, delphinidin is present in blue wheat, but not in the AnthoGrain^TM^ variety. During processing, the composition of anthocyanins in blue (Blue 2) and purple (AnthoGrain^TM^) wheats changed (Table 3, Tables S1 and S2). Some anthocyanin species were conserved, while others were lost during mechanical and thermal processing. In the AnthoGrain^TM^ products, only five anthocyanin species were well enough conserved in the final product to be detected by LC-MS/MS compared to the twelve species observed in the starting wholemeal. In the Blue 2 products, only three species were observed in all samples, while nine were observed in the starting wholemeal. The anthocyanins followed the general stability trends observed in previous works, which state that glycosylation and acetylation are protective against degradation. 

### 2.2. Stability of Endogenous Wheat Anthocyanins during Bread Processing 

The TAC of the bread dough immediately after mixing, after each fermentation phase (at the punching times), and in baked bread was measured (Table 4). Similarly to crackers, there was a significant loss of anthocyanins observed during dough mixing, and baking (Table 5). Again, anthocyanin losses were different in the intermediate stages of breads prepared with purple and blue wholemeal. 

As in cracker mixing, bread dough mixing and fermentation are dynamic processes, with many changing parameters that can impact the TAC of the sample. During mixing, the inclusion of oxygen into the bread dough matrix contributes to oxidative stress [21]. The oxygen level of dough is increased through the incorporation of air, and this oxygen participates in oxidation reactions [28]. The oxygen incorporated during mixing can then be used by yeast, and endogenous redox enzymes (i.e., lipoxygenase and polyphenol oxidase) [28]. These redox enzymes have also been shown to have a degradative effect on anthocyanins [21]. Purple wholemeal bread dough experienced significantly larger losses in anthocyanin content than blue wholemeal dough did during mixing. Purple wheat contains anthocyanins in the pericarp of the grain, and these are likely less protected than the anthocyanins in blue wheat aleurone. Eliášová et al. [14] observed a similar trend in bread made with purple and blue wheats as also in this study, bread made with purple wheat displayed greater anthocyanin losses during mixing [14].

Yeast plays a role in anthocyanin stability during fermentation. Bread dough becomes anaerobic within the first few minutes of fermentation, due to yeast respiration [28,29]. The yeast metabolism therefore removes some oxidative stress in the dough matrix. Additionally, as a by-product of respiration, yeast produce carbon dioxide, organic acids and alcohol [28,30]. The production of carbon dioxide and organic acids lowers the pH of the dough matrix, which has a further protective effect on anthocyanins. Ethanol is often used as a solvent for anthocyanin extraction, so its presence over this time period may begin to extract anthocyanins from within the bran matrix and solubilize them over the (acidified) dough liquor. These processes, i.e., rapid oxygen depletion, acidification of the dough matrix and dough liquor, could explain why there is an extremely low rate of anthocyanin loss during fermentation of both purple and blue wholemeal dough.

**Figure 2:**
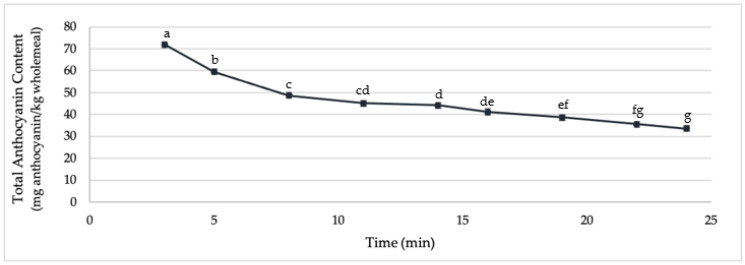
Total anthocyanin content (in mg anthocyanin/kg wholemeal) of dough/bread made from AnthoGrain™ wholemeal, baked for 3, 5, 8, 11, 14, 16.5, 19, 22, and 24 minutes. (Samples with different letters are significantly different at α = 0.05).

The baking process, however, causes significant thermal stress to the anthocyanins in bread made from both purple and blue wheats. Losses between 46–55% of anthocyanins can be observed during the conversion from bread dough to bread. Blue wholemeal bread was more affected (average loss: 54.32%) than purple wholemeal loaves (46.64%). This does not only align with the cracker results; it also aligns with previous research showing that blue wholemeal bread suffered greater anthocyanin losses during baking [14]. Bread made from coloured wheat lost on average 54% of TAC during baking. Figure 2 shows the progressive decrease in TAC during the baking process of purple wholemeal bread.

The combined effect of mixing, fermentation, and baking must also be considered. Eliášová et al. [14] found that bread made with blue wheat had greater losses during the overall breadmaking process than bread made from purple wheat. The here presented results do not align with these findings, despite alignment in the intermediate steps [14]. Rather, the here presented results better aligned with results from Bartl et al. [13], who also observed lower rates of anthocyanin loss in blue wheat bread than in purple wheat bread. As has been stated previously, anthocyanins with different functional groups and structures have different stability to the applied stresses, although these distinctions have not been fully studied or identified till date. Therefore, in addition to expected variations between purple and blue wheat varieties in anthocyanin response to stress (due to anthocyanin location [pericarp vs. aleurone]), differences in the anthocyanin content and composition of coloured wheats could also result in differences in the TAC of both intermediate processing steps, and the final bread product. Although on average, blue wholemeal reacted differently to bread processing than purple wholemeal samples, one blue wheat sample (Blue Wheat 2) was distinct from the other blue varieties tested. Despite these observed differences in their reaction to processing, HPLC chromatograms of the wholemeal samples showed that the blue wheat varieties have almost identical profiles. Therefore, this difference might be due to other disparities in starch, fibre, and protein content. The anthocyanin losses observed in bread dough and bread made from ‘Blue Wheat 2’ were more similar to those observed for AnthoGrain™ bread dough and bread.

The anthocyanin composition also changes during the processing of purple and blue wheats into bread. However, in contrast to cracker processing where the anthocyanins were either conserved or destroyed during processing, in bread, distinct anthocyanin species were observed in the bread dough and final baked bread that were not present in the original wholemeal (Table 6, Tables S1 and S2). As with crackers, AnthoGrain^TM^ bread & bread dough retained anthocyanins throughout processing. This leads to similar profiles throughout processing time. As expected, in most cases, more stable species (especially those that were more methylated and acetylated) were conserved. In the Blue Wheat 2 samples only two anthocyanin species were the same between the wholemeal and baked bread.

### 2.3. Comparison of Anthocyanin Stability in Different Cereal Processing Conditions

**Figure 3:**
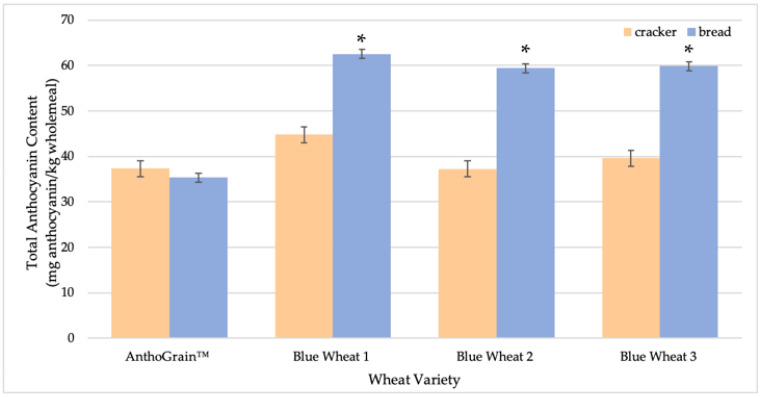
Total anthocyanin content (TAC, in mg anthocyanin/kg wholemeal) of bread and crackers made from AnthoGrain™ wholemeal, and wholemeal from Blue Wheats 1, 2, & 3. * indicates a significant difference between the TAC in cracker and bread products per wholemeal type.

As was discussed in previous sections, anthocyanin loss varied in response to the type of cereal processing applied. Despite the use of the same starting material, crackers and bread had different anthocyanin contents and compositions in the final product (Figure 3). Blue wheat retained significantly more anthocyanins in bread than in crackers, whereas purple wheat crackers and bread had no significant difference in the anthocyanin content after processing.

While the overarching production scheme of both bread and cracker production can be broken down into the general scheme of (i) dough mixing, (ii) dough shaping and resting, and (iii) baking, the conditions/parameters within each of these stages differ largely between crackers and bread (Table 7). Process parameters such as time, temperature, leavening systems, pH and moisture contents were theorized to impact the TAC of the final product.

Anthocyanins are sensitive to elevated temperatures, as well as exposure to moisture, oxygen, and high pH environments. These environmental stresses differ drastically in these two products. For example, during the fermentation of bread dough, the oxygen incorporated during mixing is rapidly depleted by yeast creating an anoxic environment. In cracker dough, a chemical leavening system is utilized, so there is no external biological system to influence the oxygen level of the dough. The differences between these environmental stresses results in the discrepancies in measured TAC of the cracker and bread samples throughout processing. Manipulation of the processes to alter these key stresses may result in decreased anthocyanin losses in the final product. This was explored to some extent via modifying the leavening system in crackers, and the baking time-temperature profile of bread.

Chemical leavening systems affect the pH of cracker dough in addition to providing necessary CO_2_ formation during baking. AnthoGrain™ wholemeal was used to make crackers with the control formulation (SAPP-40 + sodium bicarbonate), as well as samples without sodium bicarbonate, and without sodium bicarbonate and SAPP-40. Both parts of the chemical leavening system have an effect on the pH of the system (Table 8). The presence/absence of leavening agents had no significant effect on the TAC during mixing (t = 10 min). However, at most points in the process, the TAC of the modified crackers was significantly higher than the control formulation (with the exception of the no sodium bicarbonate/SAPP-40 dough at 30 min). While anthocyanins are more stable under acidic conditions, the removal of basic leavening agents did not significantly impact the TAC retention of the final cracker product. However, the modifications to the formula did impact the time point at which TAC decreased. The lower pH of the cracker dough was protecting anthocyanin against degradation during processing, but did not sufficiently mitigate the ensuing thermal degradation. Acidulants have been shown previously to have a protective effect on the anthocyanin content of cookies produced from blue maize. Li et al. [18], found that incorporating citric acid to reach a dough pH of 3.8 was ideal for the retention of anthocyanins in the final cookie. Since the cracker formulation with no baking soda resulted in an insignificantly higher TAC in the final cracker than the other two samples, future work should attempt to further reduce the pH of the dough, to potentially achieve the same results as previous work while keeping a close eye on the sensory properties of the product [18].

The baking time-temperature profile has previously been shown to have an effect on the TAC of breads made with coloured wheat wholemeal [12,13,14,15]. Previous studies have reported that anthocyanin degradation is lower during high temperature, short time processes than in those processes characterized by a lower baking temperature for a longer time [12,13,14,15]. This trend was also observed in this study on bread baked at 215 °C and 240 °C (Table 9). Anthocyanin degradation demonstrates first order kinetics, or reduced thermal stability in isothermal systems [15]. In non-isothermal systems, decreased exposure to high temperatures reduces anthocyanin degradation [12,13,14,15]. While the increased temperature would be more damaging to the anthocyanins present in the bread, the faster cooking time lowers anthocyanin exposure to temperature, and therefore this type of baking is more protective of anthocyanins. Bread baked at 240 °C for 20 min retained more anthocyanins than bread baked at 215 °C for 24 min. The selection of appropriate time-temperature combinations can minimize anthocyanin loss, and contribute to a better nutritional profile of the final product.

## 3. Materials and Methods

### 3.1. Materials

The commercial purple wheat variety AnthoGrain™, was grown in 2019 in Saskatchewan and was kindly donated by InfraReady Products (1998) Limited (Saskatoon, Saskachewan). Three experimental blue wheat varieties, i.e., 24 EA-17-1605 (Blue 1), 25 EA-17-1626 (Blue 2), and 26 EA-17-1692 (Blue 3) were grown in 2019. The experimental wheat varieties were available in limited quantities which precluded some data collection. The wheat kernels were ground using a Cyclone sample mill (Udy Co., Fort Collins, CO, USA) equipped with a 500 μm screen to produce wholemeal. The wholemeal was mixed to ensure uniformity, and kept at 4 °C until further processing. Other cracker and bread ingredients were purchased from local grocery stores. All other chemicals, reagents, and solvents were purchased from Fisher Scientific (Mississauga, ON, Canada).

### 3.2. Production of Crackers

The moisture content of the wholemeal was measured with an MB45 Moisture Analyzer (Ohaus, Switzerland), and the cracker formulation used is outlined in Table 10.

Wholemeal, and dry ingredients (sucrose, salt, SAPP-40, sodium bicarbonate) were weighed and then sifted into the bowl of a mixer (Globe, Dayton, OH, USA). Variations in the cracker recipe were tested by omitting: (1) sodium bicarbonate, and (2) both sodium bicarbonate and SAPP-40. Water, oil, and corn syrup were weighed into a separate container, stirred to disperse, and then added to the bowl of the mixer. Using the paddle attachment on speed 2, the cracker dough was mixed for 10 min. The dough was then formed into a ball, and allowed to rest at room temperature for 10 min. Using a rolling pin, the dough was rolled flat 3 times to about 1 cm in thickness, folding the dough over on itself each time (lapping). The large dough mass was then separated into three smaller, uniform samples, and allowed to rest for an additional 10 min. Each small piece of dough was then fed through a KitchenAid pasta sheeter (KitchenAid, Mississauga, ON, Canada) 3 times on setting 2 followed by an additional 3 times on setting 3 (<2 mm thick). The dough sheet was then placed on the counter, and square crackers were cut (1.5 cm × 1.5 cm), and docked using a fork. Crackers from all three dough pieces were placed on a perforated baking sheet, and baked at 249 °C for 2.5 min in a revolving oven (LC Bakery Equipment, Brantford, ON, Canada).

For samples made from AnthoGrain™ wholemeal, cracker dough samples were collected every 1.5 min during the mixing process, and after each resting stage. For samples made with blue wheat, samples of cracker dough were only collected before (t = 40 min) and after baking, due to limitations in wholemeal availability (due to experimental variety yields). The cracker dough samples were stored immediately in a −80 °C freezer to halt further chemical processes. Samples were subsequently freeze-dried (Virtis Genesis 25ES Lyophilizer, SP Scientific, Gardiner, NY, USA). Freeze-dried samples were then stored in sealed bags at room temperature until further analysis.

### 3.3. Production of Bread

The water absorption of the wholemeal was determined according to the AACC-I Approved Method 54-21.02, using a Brabender Farinograph-E (Brabender GmbH & Co. KG, Duisburg, Germany), equipped with a 50 g mixing bowl. The moisture content of the wholemeal was measured with an MB45 Moisture Analyzer (Ohaus, Switzerland). Bread samples were produced according to the AACC-I Approved optimized straight dough breadmaking method (AACC-I 10-10.03), with a 90 min fermentation time, and the recipe outlined in Table 11.

Dough mixing was performed with a 100 g pin mixer (National Manufacturing, Lincoln, Dearborn, MI, USA) for 2.0 min at room temperature. After mixing, the dough was placed in a bowl covered with a moist cloth, and fermented in a fermentation cabinet at 30 °C and 85% RH. The dough was punched after 52 (gap setting 188), 77 (gap setting 188) and 90 (gap setting 312) min of fermentation with a dough sheeter (National Manufacturing, Lincoln, USA). The fermented dough was then moulded into a loaf shape, placed into a greased metal baking tin, and fermented/proofed for an additional 33 min (123 min fermentation total). Finally, the proofed dough was baked in a rotary oven at 215 °C for 24 min. Samples were collected after 0, 52, 77, 90, and 123 min of fermentation, and after baking. For AnthoGrain™ wholemeal, samples of bread were also collected every 2.5 min during baking (215 °C) for 24 min. Bread loaves were cut into 2 cm slices after cooling to room temperature. Samples were immediately frozen in a −80 °C freezer to halt further chemical reactions. Samples were subsequently freeze-dried (Virtis Genesis 25ES Lyophilizer, SP Scientific, Gardiner, NY, USA). Freeze-dried samples were stored at room temperature in sealed bags until further analysis.

### 3.4. Anthocyanin Extraction

Freeze-dried dough pieces and bread slices from the center of the loaf were ground (including crumb and crust), with a mortar and pestle, and then a homogenous sample was taken from the ground material for further analysis. All crackers from one batch were also ground using a mortar and pestle, and a homogenous sample was taken for further analysis. The homogenous, ground material (1.00 g) was weighed into a centrifuge tube, and 10.00 mL of acidified methanol (85:15 methanol:1.00 M HCl) was added. The mixture was shaken for 30 min and centrifuged at 11,419× *g* for 30 min (Allegra X-15R centrifuge Beckman Coulter Inc., Indianapolis, IN, USA). The supernatant was decanted into a 10 mL volumetric flask, and the volume was adjusted to 10.0 mL with acidified methanol.

### 3.5. Anthocyanin Quantification

The TAC in grain samples was determined using the spectrophotometric method previously described by Abdel-Aal and Hucl [27]. In short, the extracts were centrifuged one more time to remove turbidity before the absorbance was measured. 1.50 mL of the extracts was centrifuged at 13,000× *g* for 15 min (AccuSpin Micro 17, Fisher Scientific, Ottawa, ON, Canada). The absorbance of the samples was measured on a UV/vis spectrophotometer (Genesys 180, Thermo Fisher Scientific, Waltham, MA, USA) at 535 nm. The TAC in mg anthocyanin/kg wheat material was calculated as follows:

$$TAC=\left(\frac{A}{\varepsilon }\right)\times \left(\frac{V}{1000}\right)\times \left(MW\right)\times \left(\frac{1}{SWT}\right)\times \left(10^{6}\right)$$

where A is the absorbance measured at 535 nm, ε is the molar absorptivity (for cyanidin-3-glucoside, *ε* $=25,965{\;\mathrm{cm}}^{-1}M^{-1}$), V is the total volume of anthocyanin extract (in mL), SWT is the sample weight, and *MW* is the molecular weight (for cyanidin-3-glucoside, $MW=449{\;\mathrm{gmol}}^{-1}$). This value was standardized to mg anthocyanin/kg whole kernel weight using a mass balance equation, and assuming that the other cracker/bread ingredients contributed no anthocyanins to the final *TAC*.

### 3.6. Anthocyanin Identification

Samples were screened using a validated HPLC-UV/Vis method. The three blue wheat varieties showed similar profiles using this method, and so Blue Wheat 2 was selected as a sample for identification, along with AnthoGrain^TM^. Wholemeal, cracker dough, cracker, bread dough (fermented for 77 min), and bread samples were tested. The other blue wheat samples were not tested due to low sample availability. Liquid chromatography–mass spectrometry analyses were performed on an Agilent 1200 HPLC liquid chromatograph interfaced with an Agilent UHD 6530 Q-Tof mass spectrometer at the Mass Spectrometry Facility of the Advanced Analysis Centre, University of Guelph. A C18 column (Agilent Poroshell 120, 150 mm × 4.6 mm 2.7 µm) was used for chromatographic separation with the following mobile phases: water with 0.1% formic acid (A) and acetonitrile with 0.1% formic acid (B). The mobile phase gradient was as follows: initial conditions were 5% B, hold for 3 min then increasing to 100% B in 28 min followed by a column wash at 100% B for 2 min and 10 min re-equilibration. The flow rate was maintained at 0.4 mL/min. The mass spectrometer electrospray capillary voltage was maintained at 4.0 kV and the drying gas temperature at 250 °C with a flow rate of 8 L/min. Nebulizer pressure was 30 psi and the fragmentor was set to 160. Nitrogen was used as both nebulizing and drying gas, and collision-induced gas. The mass-to-charge ratio was scanned across the *m/z* range of 300–2000 *m/z* in 4 GHz (extended dynamic range in both positive ion MS mode. The instrument was externally calibrated with the ESI TuneMix (Agilent). The sample injection volume was 5 µL. Chromatographic data including the retention times, *m/z* values (for molecular and fragment ions) and tentative identifications are included as Supplementary Materials.

### 3.7. Statistical Analysis

All experiments were carried out in triplicate, and the results are displayed as average values ± standard deviation (SD). Data were subjected to a one-way ANOVA test, and a *p*-value < 0.05 indicated a significant difference (α = 0.05). If the *p*-value was found to be <0.05 for a dataset, Tukey’s HSD testing was used to determine the statistical difference between the data in the set. A paired-sample *t*-test was used to determine significance (*p*-value < 0.05) in the difference between crackers and bread made from one wholemeal variety.

## 4. Conclusions

Processing steps that added additional oxidative or pH stress to the product (i.e., mixing), and those which added thermal stress (e.g., baking), had larger impacts on the TAC than the other processing steps. In both cracker and bread production, the resting/fermentation step was shown to have little to no impact on the TAC of the resulting product.

Products made from blue wheat varieties retained more total anthocyanins after processing than products made from purple wheat. Proportionally however, the anthocyanin retention rate was similar for blue and purple wheats in bread and crackers. Two blue wheat samples (Blue Wheat 1, and Blue Wheat 3) were more sensitive to thermal degradation of the anthocyanins (due to baking), and less susceptible to oxidative and pH stresses (due to mixing) than were the AnthoGrain™ and Blue Wheat 2 samples. The causes of the observed differences in sensitivity need to be further investigated but could potentially be associated with the different localizations of the anthocyanins over the kernel tissue (pericarp vs. aleurone). The anthocyanin composition of the products was different depending on the starting composition, indicating that the starting composition may play an important role in the stability of the final product.

The differing processing parameters and steps involved in the production of crackers and bread result in different final TAC. While the TAC of blue wholemeal bread was higher than that found for blue wholemeal crackers, purple wholemeal bread and crackers had similar final TAC. Changes to the leavening system in crackers (to decrease the pH), and to the baking time-temperature profile of bread (high temperature, short time) resulted in decreased anthocyanin losses.

The role of anthocyanin localization in the starting material should be further studied, in order to elucidate its role in anthocyanin retention during regular cereal processing. Additionally, modifications to the processing parameters of cereal products using coloured wheat wholemeal should be further explored to minimize anthocyanin losses throughout processing. However, this study provides essential insight into the processing steps which cause anthocyanin degradation, and investigates preliminary strategies to combat these losses.

## Figures and Tables

**Table 1:** Total anthocyanin content (mg anthocyanin/kg wholemeal) of AnthoGrain™ and Blue (1, 2, 3) wheat wholemeal, cracker dough (40 min) just prior to baking, and baked crackers.

Time Point	AnthoGrain™	Blue 1	Blue 2	Blue 3
Wholemeal	121.19 ± 4.77 a	168.62 ± 10.50 a	194.36 ± 5.51 a	135.64 ± 6.95 a
Cracker Dough (40 min)	54.30 ± 1.19 b	94.77 ± 1.60 b	89.14 ± 0.60 b	87.13 ± 0.44 b
Baked Cracker	37.31 ± 0.72 c	44.76 ± 3.29 c	37.22 ± 1.15 c	39.61 ± 0.87 c

Samples in the same column with different letters are significantly different at α = 0.05.

**Table 2:** Loss of total anthocyanin content (expressed as %) at each cracker processing step.

Process.	AnthoGrain™	Blue 1	Blue 2	Blue 3
Mixing ^+^	52.34 ± 0.92 d	n.d.	n.d.	n.d.
Resting ^++^	5.87 ± 4.56 a	n.d.	n.d.	n.d.
Mixing and Resting ^+^	n.d.	43.64 ± 3.91 c	54.11 ± 1.51 d	35.65 ± 3.26 b
Baking ^++^	31.06 ± 2.80 b	52.79 ± 3.00 d	58.25 ± 1.25 d	54.55 ± 0.92 d
Overall ^+^	69.11 ± 0.72 e	73.31 ± 3.51 ef	80.83 ± 1.13 f	70.73 ± 2.00 e

n.d. not determined due to low availability of the wheat variety. Samples with different letters are significantly different at α = 0.05. ^+^ Calculated using the wholemeal total anthocyanin content as 100%. ^++^ Calculated using the total anthocyanin content of the previous step as 100%.

**Table 3:** Anthocyanin species in AnthoGrain^TM^ and Blue 2 wholemeal, cracker dough, and baked crackers as detected and identified by LC-MS/MS analysis.

	**Wholemeal**		**Cracker Dough**		**Baked Cracker**	
	Aglycone	Substituent	Aglycone	Substituent	Aglycone	Substituent
Antho Grain^TM^	Cyanidin	Acetylated hexose				
	Cyanidin	Hexose+ malonic acid	Cyanidin	Hexose+ malonic acid		
			Cyanidin	Hexose		
					Cyanidin	
	Peonidin	Hexose	Peonidin	Hexose	Peonidin	Hexose
	Peonidin	Hexose+ malonic acid	Peonidin	Hexose+ malonic acid	Peonidin	Hexose+ malonic acid
	Peonidin	Acetylated hexose				
	Peonidin	Succinyl hexose				
	Peonidin	Hexose+ malonic acid+ acetic acid				
	Peonidin	Hexose+ deoxy hexose	Peonidin	Hexose+ deoxy hexose	Peonidin	Hexose+ deoxy hexose
	Peonidin	Hexose				
	Peonidin		Peonidin		Peonidin	
	Malvidin	Hexose+ deoxy hexose			Malvidin	Hexose+ deoxy hexose
	Malvidin		Malvidin		Malvidin	
Blue 2	Cyanidin	Hexose+ deoxy hexose	Cyanidin	Hexose+ hexose		
			Cyanidin	Hexose+ deoxy hexose	Cyanidin	Hexose+ deoxy hexose
	Delphinidin	Hexose+ deoxy hexose	Delphinidin	Hexose+ deoxy hexose	Delphinidin	Hexose+ deoxy hexose
	Delphinidin	Hexose	Delphinidin	Hexose		
	Delphinidin	Acetylated hexose	Delphinidin	Hexose+ acetic acid		
					
	Peonidin	Hexose+ deoxy hexose+ hexose	Peonidin	Hexose+ deoxy hexose+ hexose	Peonidin	Hexose+ deoxy hexose+ hexose
	Peonidin	Hexose+ hexose				
	Peonidin	Hexose+ deoxy hexose	Peonidin	Hexose+ deoxy hexose		
	Malvidin	Hexose+ hexose				
	Malvidin	Hexose+ deoxy hexose	Malvidin	Hexose+ deoxy hexose	Malvidin	Hexose+ deoxy hexose

**Table 4:** Total anthocyanin content (mg anthocyanin/kg wholemeal) of AnthoGrain™ and Blue (1, 2, 3) wheat wholemeal, bread dough (0 min), fermented dough (52–123 min), and baked bread.

Time Point	AnthoGrain™	Blue 1	Blue 2	Blue 3
Wholemeal	121.19 ± 4.77 a	168.62 ± 10.50 a	194.36 ± 5.51 a	135.64 ± 6.95 a
Dough (0 min)	75.22 ± 0.24 b	141.28 ± 0.81 b	125.57 ± 1.76 b	125.48 ± 2.04 b
Fermented Dough (52 min)	73.31 ± 1.17 bc	139.87 ± 1.73 b	124.98 ± 1.86 b	124.93 ± 0.61 b
Fermented Dough (77 min)	71.99 ± 1.05 c	137.035 ± 0.42 b	126.90 ± 4.20 b	122.82 ± 3.15 b
Fermented Dough (90 min)	71.89 ± 0.26 c	138.26 ± 1.68 b	129.00 ± 0.78 b	124.27 ± 2.21 b
Fermented Dough (123 min)	71.79 ± 0.90 c	141.56 ± 1.29 b	131.06 ± 1.58 b	125.40 ± 0.56 b
Bread	35.53 ± 0.35 d	62.56 ± 0.31 c	59.38 ± 0.47 c	59.86 ± 2.31 c

Samples in the same column with different letters are significantly different at α = 0.05.

**Table 5:** Loss of total anthocyanin content (expressed as %) at each bread processing step.

Process	AnthoGrain™	Blue 1	Blue 2	Blue 3
Mixing ^+^	38.01 ± 2.81 d	16.01 ± 4.90 c	35.35 ± 2.45 d	7.12 ± 6.10 b
Fermentation ^++^	4.56 ± 1.45 b	−0.20 ± 1.48 ab	−4.38 ± 0.23 a	1.15 ± 0.45 ab
Baking ^++^	46.64 ± 3.40 e	55.81 ± 0.56 fg	54.69 ± 0.55 ef	52.26 ± 1.85 ef
Overall ^+^	68.35 ± 2.63 e	62.81 ± 2.31 ge	69.44 ± 0.73 e	55.81 ± 2.27 fg

Samples with different letters are significantly different at α = 0.05. ^+^ Calculated using the wholemeal total anthocyanin content as 100%. ^++^ Calculated using the total anthocyanin content of the previous step as 100%.

**Table 6:** Anthocyanin species in AnthoGrain^TM^ and Blue 2 wholemeal, bread dough (77min), and baked bread from LC-MS/MS analysis.

	**Wholemeal**		**Bread Dough**		**Bread**	
	Aglycone	Substituent	Aglycone	Substituent	Aglycone	Substituent
Antho Grain^TM^	Cyanidin	Acetylated hexose				
	Cyanidin	Hexose+ malonic acid	Cyanidin	Hexose+ malonic acid		
			Cyanidin	Hexose	Cyanidin	Hexose
					Cyanidin	
	Peonidin	Hexose	Peonidin	Hexose	Peonidin	Hexose
	Peonidin	Hexose+ malonic acid	Peonidin	Hexose+ malonic acid	Peonidin	Hexose+ malonic acid
	Peonidin	Acetylated hexose				
	Peonidin	Succinyl hexose				
	Peonidin	Hexose+ malonic acid+ acetic acid				
	Peonidin	Hexose+ deoxy hexose	Peonidin	Hexose+ deoxy hexose		
	Peonidin	Hexose				
	Peonidin		Peonidin		Peonidin	
					Peonidin	Deoxy hexose+ formic acid
	Malvidin	Hexose+ deoxy hexose				
	Malvidin		Malvidin		Malvidin	
Blue 2	Cyanidin	Hexose+ deoxy hexose			Cyanidin	Hexose+ deoxy hexose
					
					Cyanidin	Hexose
	Delphinidin	Hexose+ deoxy hexose			Delphinidin	Hexose+ deoxy hexose
	Delphinidin	Hexose	Delphinidin	Hexose	Delphinidin	Hexose
	Delphinidin	Acetylated hexose				
			Delphinidin	Di-rutinose		
	Peonidin	Hexose+ deoxy hexose+ hexose				
	Peonidin	Hexose+ hexose	Peonidin	Hexose+ hexose		
	Peonidin	Hexose+ deoxy hexose			Peonidin	Hexose+ deoxy hexose
			Peonidin	Di-rutinose		
					Peonidin	Deoxy hexose+ Formic acid
	Malvidin	Hexose+ hexose				
	Malvidin	Hexose+ deoxy hexose			Malvidin	Hexose+ deoxy hexose

**Table 7:** Process parameters for the production of crackers and bread.

Process	Cracker	Bread
Process Time	Short (45 min)	Long (147 min)
Baking		
Temperature	High (249 °C)	Low (205 °C)
Time	3 min	24 min
Leavening System	Chemical	Biological
Dough pH	7.0	5.45
Moisture	Low	High
	46% (dough)	59% (dough)
	2% (baked product)	35% (baked product)

**Table 8:** Total anthocyanin content (mg anthocyanin/kg wholemeal) of cracker dough (at various resting points) and crackers made from AnthoGrain™ wholemeal (control), without sodium bicarbonate, and without sodium bicarbonate and SAPP-40.

Time Point	Control	No Sodium Bicarbonate	No Sodium Bicarbonate No SAPP-40
	pH-6.96	5.24	5.77
Wholemeal	121.19 ± 4.77 a	121.19 ± 4.77 a	121.19 ± 4.77 a
10 min	66.70 ± 2.95 bcd	69.79 ± 0.93 bc	68.04 ± 1.43 bc
20 min	56.88 ± 0.33 e	68.48 ± 0.75 bc	65.20 ± 2.71 cd
30 min	56.02 ± 0.44 e	69.87 ± 3.32 bc	58.45 ± 0.54 e
40 min	54.30 ± 1.19 e	69.97 ± 0.17 b	63.21 ± 0.30 d
Baked Cracker	37.41 ± 0.72 fg	42.00 ± 1.24 f	36.03 ± 1.29 g

Samples with different letters are significantly different at α = 0.05.

**Table 9:** Total anthocyanin content (mg anthocyanin/kg wholemeal) of bread made from AnthoGrain™ wholemeal baked at 215 °C and 240 °C.

Temperature (°C)	Time (min)	Total Anthocyanin Content
215	24	38.99 ± 2.18 a
240	20	56.18 ± 4.81 b

Samples with different letters are significantly different at α = 0.05.

**Table 10:** Cracker formulation.

Ingredients	Weight (g)	Percentage (% *w/w*)
Wholemeal (13.0% moisture)	100	60.24
Water	40	24.10
Oil	12	7.23
Corn Syrup	4	2.41
Sucrose	6	3.61
Salt (NaCl)	1.5	0.90
Sodium Bicarbonate	1.25	0.75
Sodium Acid Pyrophosphate 40 (SAPP-40)	1.25	0.75

**Table 11:** Bread formulation.

Ingredients	Weight (g)	Percentage (% *w/w*)
Wholemeal (14.0% moisture)	100	55.93
Water	63	35.23
Yeast	5.3	2.96
Sucrose	6	3.36
Salt (NaCl)	1.5	0.84
Shortening	3	1.68

## Data Availability

The data presented in this study are available on request from the corresponding author.

## Supplementary Materials

The following supporting information can be downloaded at: https://www.mdpi.com/article/10.3390/molecules27217180/s1, Table S1: Chromatographic data including retention time, *m*/*z* for M+ and fragments, and tentative identifications) for the anthocyanin species in AnthoGrainTM wholemeal, cracker dough, and baked crackers, bread dough, and baked bread as detected and identified by LC-MS/MS analysis. Table S2: Chromatographic data (including retention time, *m/z* for M+ and fragments, and tentative identifications) for the anthocyanin species in Blue Wheat 2 wholemeal, cracker dough, and baked crackers, bread dough and baked bread as detected and identified by LC-MS/MS analysis.

## Sample Availability

Samples of the compounds are not available from the authors.

## References

[1] Abdel-Aal E.S.M., Awika J.M., Piironen V., Bean S. (2011). Anthocyanin-Pigmented Grain Products. Advances in Cereal Science: Implications to Food Processing and Health Promotion.

[2] Arendt E.K., Zannini E. (2013). Cereal Grains for the Food and Beverage Industries.

[3] Moore J., Hao J. (2012). Antioxidant and Health Promoting Properties of Wheat (*Triticum* spp.). Cereals Pulses Nutraceutical Prop. Health Benefits.

[4] Gani A., Wani S.M., Masoodi F.A., Hameed G. (2012). Whole-Grain Cereal Bioactive Compounds and Their Health Benefits: A Review. J. Food Process. Technol..

[5] Fardet A., Rock E., Rémésy C. (2008). Is the in Vitro Antioxidant Potential of Whole-Grain Cereals and Cereal Products Well Reflected in Vivo?. J. Cereal Sci..

[6] Francavilla A., Joye I.J. (2020). Anthocyanins in Whole Grain Cereals and Their Potential Effect on Health. Nutrients.

[7] Castañeda-Ovando A., Pacheco-Hernández M.D.L., Páez-Hernández M.E., Rodríguez J.A., Galán-Vidal C.A. (2009). Chemical Studies of Anthocyanins: A Review. Food Chem..

[8] Rodriguez-Amaya D.B. (2019). Update on Natural Food Pigments—A Mini-Review on Carotenoids, Anthocyanins, and Betalains. Food Res. Int..

[9] Damodoran S., Parkin K., Fennema O.R., Srinivasan D., Parkin K.L. (2009). Fennema’s Food Chemistry.

[10] Knievel D.C., Abdel-Aal E.S.M., Rabalski I., Nakamura T., Hucl P. (2009). Grain Color Development and the Inheritance of High Anthocyanin Blue Aleurone and Purple Pericarp in Spring Wheat (*Triticum Aestivum* L.). J. Cereal Sci..

[11] Blesso C.N. (2019). Dietary Anthocyanins and Human Health. Nutrients.

[12] Seo Y., Moon Y., Kweon M. (2021). Effect of Purple-Colored Wheat Bran Addition on Quality and Antioxidant Property of Bread and Optimization of Bread-Making Conditions. Appl. Sci..

[13] Bartl P., Albreht A., Skrt M., Tremlova B., Ostadlova M., Smejkal K., Vovk I., Ulrih N.P.N.P., Tremlová B., Oš͗ádalová M. (2015). Anthocyanins in Purple and Blue Wheat Grains and in Resulting Bread: Quantity, Composition, and Thermal Stability. Int. J. Food Sci. Nutr..

[14] Eliášová M., Kotíková Z., Lachman J., Orsák M., Martinek P. (2020). Influence of Baking on Anthocyanin Content in Coloured-Grain Wheat Bread. Plant Soil Environ..

[15] Sui X., Yap P.Y., Zhou W. (2015). Anthocyanins During Baking: Their Degradation Kinetics and Impacts on Color and Antioxidant Capacity of Bread. Food Bioprocess Technol..

[16] Yu L., Beta T. (2015). Identification and Antioxidant Properties of Phenolic Compounds during Production of Bread from Purple Wheat Grains. Molecules.

[17] Li W., Pickard M.D., Beta T. (2007). Food Chemistry Effect of Thermal Processing on Antioxidant Properties of Purple Wheat Bran. Food Chem..

[18] Li J., Walker C.E., Faubion J.M. (2011). Acidulant and Oven Type Affect Total Anthocyanin Content of Blue Corn Cookies. J. Sci. Food Agric..

[19] Ficco D.B.M., de Simone V., de Leonardis A.M., Giovanniello V., del Nobile M.A., Padalino L., Lecce L., Borrelli G.M., de Vita P. (2016). Use of Purple Durum Wheat to Produce Naturally Functional Fresh and Dry Pasta. Food Chem..

[20] Davidson I. (2019). Dough Mixing. Biscuit, Cookie, and Cracker Production: Process, Production, and Packaging Equipment.

[21] Patras A., Brunton N.P., O’Donnell C., Tiwari B.K. (2010). Effect of Thermal Processing on Anthocyanin Stability in Foods; Mechanisms and Kinetics of Degradation. Trends Food Sci. Technol..

[22] Brauch J.E., Kroner M., Schweiggert R.M., Carle R. (2015). Studies into the Stability of 3-O-Glycosylated and 3,5-O-Diglycosylated Anthocyanins in Differently Purified Liquid and Dried Maqui (Aristotelia Chilensis (Mol.) Stuntz) Preparations during Storage and Thermal Treatment. J. Agric. Food Chem..

[23] Abdel-Aal E.S.M., Hucl P., Shipp J., Rabalski I. (2016). Compositional Differences in Anthocyanins from Blue- and Purple-Grained Spring Wheat Grown in Four Environments in Central Saskatchewan. Cereal Chem..

[24] Abdel-Aal E.S.M., Hucl P. (2003). Composition and Stability of Anthocyanins in Blue-Grained Wheat. J. Agric. Food Chem..

[25] Abdel-Aal E.S.M., Young J.C., Rabalski I. (2006). Anthocyanin Composition in Black, Blue, Pink, Purple, and Red Cereal Grains. J. Agric. Food Chem..

[26] Varga M., Bánhidy J., Cseuz L., Matuz J. (2013). The Anthocyanin Content of Blue and Purple Coloured Wheat Cultivars and Their Hybrid Generations. Cereal Res. Commun..

[27] Abdel-Aal E.S.M., Hucl P. (1999). A Rapid Method for Quantifying Total Anthocyanins in Blue Aleurone and Purple Pericarp Wheats. Cereal Chem..

[28] Decamps K., Joye I.J., de Vos D.E., Courtin C.M., Delcour J.A. (2016). Molecular Oxygen and Reactive Oxygen Species in Bread-Making Processes: Scarce, but Nevertheless Important. Crit. Rev. Food Sci. Nutr..

[29] Joye I.J., Draganski A., Delcour J.A., Ludescher R.D. (2012). Monitoring Molecular Oxygen Depletion in Wheat Flour Dough Using Erythrosin B Phosphorescence: A Biophysical Approach. Food Biophys..

[30] Jayaram V.B., Cuyvers S., Lagrain B., Verstrepen K.J., Delcour J.A., Courtin C.M. (2013). Mapping of Saccharomyces Cerevisiae Metabolites in Fermenting Wheat Straight-Dough Reveals Succinic Acid as PH-Determining Factor. Food Chem..

