# Supplementary material for: Anthocyanin Content of Crackers and Bread Made with Purple and Blue Wheat Varieties
Source: Molecules. 2022 Oct 24;27(21):7180. doi: 10.3390/molecules27217180 (PMC9656245; doi:10.3390/molecules27217180)
Supplement: Supplementary file 1 [file molecules-27-07180-s001.zip › molecules-1934080-supplementary.pdf]

Supplementary Materials

The following tables (Table S1, & Table S2) contain chromatographic data from samples analyzed using LC-MS/MS for anthocyanin composition.

Table S1. Chromatographic data (including retention time, m/z for M+ and fragments, and tentative identifications) for the anthocyanin species in AnthoGrain™ wholemeal, cracker dough, and baked crackers as detected and identified by LC-MS/MS analysis.

Retention Time	Tentative Identification		$[M+H]^+$ (m/z)	Fragment (m/z)
	Aglycone	Substituent		
		Wholemeal		
13.13	Cyanidin	Acetylated hexose	491	
13.24	Cyanidin	Hexose+ malonic acid	535	
12.92	Peonidin	Hexose	463	
13.71	Peonidin	Hexose+ malonic acid	549	
13.94	Peonidin	Acetylated hexose	505	
14.53	Peonidin	Succinyl hexose	563	547
14.80	Peonidin	Hexose+ malonic acid + acetic acid	591	
15.33	Peonidin	Hexose+ deoxy hexose	609	463
16.09	Peonidin	Hexose	463	
16.11	Peonidin		477	317
15.21	Malvidin	Hexose+ deoxy hexose	639	493
17.60	Malvidin		521	
		Cracker Dough		
12.42	Cyanidin	Hexose	449	
12.94	Peonidin	Hexose	463	374
13.22	Cyanidin	Hexose+ malonic acid	535	327
13.90	Peonidin	Hexose+ malonic acid	549	505, 355
14.28	Peonidin	Hexose+ deoxy hexose	609	463
16.24	Peonidin		477	
17.54	Malvidin		521	
		Baked Cracker		
13.03	Peonidin	Hexose	463	

13.85	Peonidin	Hexose+ malonic acid	549	499
14.18	Cyanidin		619	364
15.23	Malvidin	Hexose+ deoxy hexose	639	494
15.37	Peonidin	Hexose+ deoxy hexose	609	465, 355
16.22	Peonidin		477	
17.55	Malvidin		521	443
Bread Dough (77 min)				
12.37	Cyanidin	Hexose	449	350
13.38	Cyanidin	Hexose+ malonic acid	535	319
13.81	Peonidin	Hexose+ malonic acid	549	400
16.23	Peonidin		477	
17.43	Peonidin	Hexose+ deoxy hexose	563	385, 380
17.72	Peonidin	Hexose	491	336
19.97	Malvidin		331	
Baked Bread				
12.35	Cyanidin	Hexose	449	392
13	Peonidin	Hexose	463	
13.23	Cyanidin		488	327
13.8	Peonidin	Hexose+ malonic acid	549	389
16.11	Peonidin	Deoxy hexose+ formic acid	475	
17.66	Peonidin		491	
20.16	Malvidin		331	

Table S2. Chromatographic data (including retention time, m/z for M+ and fragments, and tentative identifications) for the anthocyanin species in Blue Wheat 2 wholemeal, cracker dough, and baked crackers as detected and identified by LC-MS/MS analysis.

Retention Time	Tentative Identification		$[M+H]^+$ (m/z)	Fragment (m/z)
	Aglycone	Substituent		
		Wholemeal		
12.20	Cyanidin	Hexose+ deoxy hexose	595	449
11.63	Delphinidin	Hexose+ deoxy hexose	611	465
11.66	Delphinidin	Hexose	465	
13.28	Delphinidin	Acetylated hexose	507	
14.76	Peonidin	Hexose+ deoxy hexose + hexose	771	207, 463, 609
14.87	Peonidin	Hexose+ hexose	625	463, 609
15.38	Peonidin	Hexose+ deoxy hexose	609	463
15.10	Malvidin	Hexose+ hexose	655	493, 347
15.23	Malvidin	Hexose+ deoxy hexose	639	493
		Cracker Dough		
10.26	Cyanidin	Hexose+ hexose	611	449, 372
11.77	Delphinidin	Hexose+ deoxy hexose	611	465
11.8	Delphinidin	Hexose	465	
12.26	Cyanidin	Hexose+ deoxy hexose	595	450
12.74	Peonidin	Hexose+ deoxy hexose	609	355, 460
13.3	Delphinidin	Hexose+ acetic acid	367	204
14.78	Peonidin	Hexose+ deoxy hexose+ hexose	771	463, 207
15.29	Malvidin	Hexose+ deoxy hexose	639	493
		Baked Cracker		
11.75	Delphinidin	Hexose+ deoxy hexose	465	
12.30	Cyanidin	Hexose+ deoxy hexose	595	410
12.97	Malvidin	Hexose+ deoxy hexose	639	394
14.76	Peonidin	Hexose+ deoxy hexose+ hexose	771	463
		Bread Dough (77 min)		
11.69	Delphinidin	Hexose	465	303
11.76	Delphinidin	Di-rutinose	611	303
15.27	Peonidin	Hexose+ hexose	609	301
17.58	Peonidin	Di-rutinose	491	301, 101

		Baked Bread		
11.79	Delphinidin	Hexose+ deoxy hexose	611	465
11.80	Delphinidin	Hexose	465	
12.24	Cyanidin	Hexose+ deoxy hexose	595	433, 339
12.35	Cyanidin	Hexose	449	188
12.95	Malvidin	Hexose+ deoxy hexose	639	394
15.38	Peonidin	Hexose+ deoxy hexose	609	463
16.16	Peonidin	Deoxy hexose+ Formic acid	475	
